# Effect of Speech Material and Scoring Method on Psychometric Curves for Cochlear Implant Users and Typical Hearing Listeners

**DOI:** 10.1097/AUD.0000000000001672

**Published:** 2025-04-29

**Authors:** Hendrik Christiaan Stronks, Robin van Deurzen, Paula Louisa Jansen, Jeroen Johannes Briaire, Johan Hubertus Maria Frijns

**Affiliations:** 1 Department of Otorhinolaryngology and Head & Neck surgery, Leiden University Medical Center, Leiden, the Netherlands; 2 Leiden Institute for Brain and Cognition, Leiden, the Netherlands; 3 Department of Bioelectronics, Delft University of Technology, Delft, the Netherlands.

**Keywords:** Cochlear implants, Digit-in-noise, Dutch, Flemish, Leuven intelligibility sentences test, Matrix test, Sensorineural hearing loss, Slope, Speech recognition threshold, SRT

## Abstract

**Objectives:**

Cochlear implants (CIs) are the primary treatment for severe-to-profound hearing loss. For CI users, speech intelligibility (SI) is often excellent in quiet yet degrades dramatically in background noise. Scientific and clinical testing of the effects of noise on SI is routinely performed with speech-in-noise tests. The sensitivity of these tests to signal to noise ratio depends on the slope of their psychometric curve. This slope is not always known for CI users, and direct comparisons between typical hearing (TH) listeners and CI users are lacking.

**Design:**

We present a comparative study of a digit test (DIN), a Matrix sentence test, and an everyday sentence test (LIST) for a group of CI users and TH listeners, with use of word (digit) and sentence (triplet) scoring in the free field. We report descriptive statistics and effect size measures of the psychometric slope and the speech reception threshold (SRT) for each speech test.

**Results:**

For CI users, the slopes of the psychometric curve were significantly shallower and SRTs significantly higher than those of TH listeners. The shallowest slope was seen with the Matrix test. However, the small variances of the slope and the SRT resulted in effect size estimates that fell between those of the other two tests. The DIN test was associated with steeply sloped psychometric curves with low variance. The scoring method did not substantially affect slopes and SRTs for the DIN test and LIST sentences, but word scoring resulted in shallow slopes and substantially worse SRTs for CI users.

**Conclusions:**

The DIN test stood out in this study as an attractive speech-in-noise test for CI users, with steep slopes and low variance in slopes and SRTs among participants. Digit and keyword scoring appear to be viable options for the DIN test and LIST sentences, respectively, potentially increasing the number of available test items. For the Matrix test, sentence scoring yielded shallow slopes and deteriorated SI, especially for the CI group. We recommend word scoring for the Dutch–Flemish Matrix test.

## INTRODUCTION

Cochlear implants (CIs) are currently the first-line treatment for severe-to-profound sensorineural hearing loss. They bypass the degenerated hair cells in the cochlea by stimulating the auditory nerve electrically (Naples & Ruckenstein 2020). CIs provide excellent speech recognition in quiet for most users, but in the presence of background noise, speech intelligibility (SI) typically drops sharply, more so than for typical hearing (TH) listeners (Cullington & Zeng 2008).

Psychometric curves offer a way to visualize the effect of noise on SI. These curves reflect SI as a function of signal to noise ratio (SNR) and are sigmoid, such as the Weibull or logistic function (Kingdom & Prins 2010). The parameters that characterize psychometric functions are threshold *α*, slope *β*, guess rate *γ*, and lapse rate *λ* (Kingdom & Prins 2010). For psychometric curves associated with listening in noise, the *α* parameter equals the SNR (sound level) at the steepest point on the curve, where the change in SI per unit of SNR is maximal. For symmetric functions, this inflection point is the SNR, where SI equals 50%, also known as the speech recognition threshold (SRT). SI measurements thus are most sensitive to SNR near the SRT (MacPherson & Akeroyd 2014). *γ* reflects the guess rate and approximates 0% in open-set tests because chance levels approach 0. This parameter can be substantially higher for closed sets. *λ* represents the maximum possible SI and typically equals 100% for TH listeners, but CI users rarely reach that level of SI, even in quiet (Stronks et al. 2021).

The features of speech-in-noise (SpiN) tests determine the shape of their psychometric curves (Steeneken 2014). Speech materials used in clinical contexts typically rely on sentences, single words, or digits. SpiN tests based on single words often yield shallow-sloping psychometric curves, so that these tests are relatively insensitive even near the SRT. An advantage of this feature is that these tests can be deployed over a wide range of SNRs (Visentin & Prodi 2018; Rönnberg et al. 2019). By contrast, SpiN tests based on sentences are characterized by steeper psychometric functions that cover a narrow range of SNRs. Items in a speech corpus are often selected based on their psychometric profiles to deliver a SpiN test with a steep slope. Although this choice results in increased sensitivity and thus reliable retrieval of the SRT with an adaptive procedure, the range of SNRs in which these curves can be deployed is limited (Visentin & Prodi 2018).

In broad terms, speech tests can be open-set or closed-set (Billings et al. 2023). Open-set tests have an unlimited number of possible responses and include the widely used sentence-in-noise tests, such as the Bamford–Kowal–Bench sentences (Bench et al. 1979) and consonant-vowel-consonant words (Bosman et al. 1992). A closed-set test consists of a finite group of items that are often known to the listener (Billings et al. 2023). Typically, a closed set contains a small number of items, and the stimuli are based on (semi-)random combinations of these items. Representative examples of closed-set speech tests are the Matrix test and the digits-in-noise (DIN) test, both of which are currently available in many languages (Kollmeier et al. 2015; Smits et al. 2016). The terms “closed set” and “open set” also sometimes are used strictly in relation to the response alternatives, so that closed set can refer to multiple-choice testing and open-set to open-ended questions (Kollmeier et al. 2015). Here, we use the definitions of Billings et al. (2023): A test is a closed set when it relies on a restricted number of speech items and is an open set when it uses many different, unique test stimuli.

Closed-set tests based on words or phonemes rely less on cognitive processes such as memory recall than everyday communication tasks that involve use of the lexical context for postdictive inference of masked words. As a result, open-set sentence tests are commonly viewed as being the most representative of realistic speech (Billings et al. 2023). In the absence of other variables, speech tests based on context-rich materials are associated with steeper psychometric slopes and lower (i.e., better) SRTs than those with little context (Kalikow et al. 1977).

The psychometric curve also is shaped by the choice of scoring method. For sentence tests, sentence scoring yields steeper slopes and lower SRTs than word scoring (Versfeld et al. 2000). Likewise, with the DIN test, triplet scoring results in steeper slopes than digit scoring for TH listeners, but no effect on the SRT has been reported (Smits & Houtgast 2006; Denys et al. 2019). Word scoring (or digit scoring for the DIN test) does have the advantage of requiring fewer stimulus presentations to reach the same reliability as the other methods because multiple items can be scored per stimulus. This benefit saves time and improves the efficiency of using available speech material, which is important for open-set material with a restricted number of items. Alternatively, word scoring can be used instead of sentence scoring to enhance the precision of SI outcomes with the same number of item presentations. Word scoring may offer an advantage because CI users rarely reach 100% SI, even in quiet (Ma et al. 2023), and sentence scoring may be too stringent for this group.

Hearing loss is associated with higher SRTs and shallower curves (Smits & Festen 2011; MacPherson & Akeroyd 2014). Consequently, speech tests administered to CI users can be expected to be less sensitive to SNR than speech tests administered to TH listeners. Because the psychometric curves of SpiN tests are usually based on testing in young TH listeners, the resulting characteristics may not be representative of CI users. Furthermore, age is negatively associated with SI in noise, even when hearing abilities are normal, presumably because of changes in cognition (Goossens et al. 2017). For these reasons, comparing CI users with age-matched TH listeners can offer a more accurate picture of how the psychometric functions associated with TH listeners translate to CI users.

In addition to psychometric characteristics, more practical factors affect the best choice of speech material for a particular application. For research on CIs and other auditory implants or assistive devices, the same speech material often must be repeatedly administered to the same participant. For speech corpora consisting of unique sentences, learning effects are substantial with multiple exposures (Yund & Woods 2010), potentially affecting outcomes. By contrast, learning effects are considered negligible (House et al. 1965) with (semi-)randomly generated closed-set materials, which thus can be used indefinitely on the same listener. Nonetheless, significant learning effects on the Matrix test have been observed among CI users, especially across test sessions (Stronks et al. 2022).

Speech rate should also be considered, especially for CI users, who generally perform better at lower speech rates and when the speech is context-rich. Slowly spoken speech material rich in context therefore can be useful for CI users and other populations with severe or profound hearing loss (Van Wieringen & Wouters 2008). Lexical complexity may be a limitation for certain groups, especially children. In these instances, closed-set speech material such as that deployed in the DIN test is preferable (Smits & Festen 2011).

To assess psychometric curves associated with CI use, we administered three different Dutch-based speech tests to a group of unilateral CI users and age-matched TH listeners. Two sentence tests—the LIST sentences test (Van Wieringen & Wouters 2008) and the Matrix test (Luts et al. 2014)—were used, along with the DIN test based on triplets of digits (Smits et al. 2013). We chose these speech tests because they each have distinct characteristics that potentially affect their psychometric curves.

The DIN test is characterized by a closed set of nine digits presented in triplets (Zokoll et al. 2012). Digit triplets lack sentence structure and contextual information. Typically, the DIN test is administered with triplet scoring, so that all three digits must be repeated correctly and in the correct order. This way of scoring increases the slope of the curve (Denys et al. 2019). DIN triplets are lexically simple, and given its small set, the DIN test is considered cognitively undemanding (Smits et al. 2013; Kaandorp et al. 2017). It is associated with a steep slope (18%·dB^−1^) (Smits et al. 2016) and a relatively low SRT (−8.8 dB SNR) for TH listeners (Smits et al. 2013).

The Matrix test (Kollmeier et al. 2015) deploys sentences of 5 words with a fixed syntactic structure (name, verb number, adjective, and noun) drawn from a closed set of 50 words. As a result, Matrix sentences are cognitively more demanding than DIN triplets because the number of items and options is greater. Matrix sentences are grammatically correct but lack semantic context and can be unpredictable (e.g., “Lucas buys ten red bicycles”). This test is usually administered using word scoring. In TH listeners, it is associated with a shallower slope (14%·dB^−1^) than DIN and a relatively low SRT (−10 dB SNR) (Luts et al. 2014).

The LIST material consists of an open set of grammatically correct sentences rich in context, such as, “The airport was closed because of the bad weather.” Because of the contextual information, LIST sentences can be considered predictable. They were developed specifically with CI users in mind and are spoken in a well-articulated manner. LIST sentences are typically administered using sentence scoring, where only a subset of keywords must be repeated correctly for the answer to be considered correct. In TH listeners, this test is characterized by a steep slope of 18%·dB^−1^ and a relatively high SRT of −8 dB SNR (Van Wieringen & Wouters 2008).

Despite the popularity of the Dutch-Flemish Matrix test in Dutch-speaking countries (Vanthornhout et al. 2018; Stronks et al. 2020, 2023; Maele et al. 2021; Drakopoulos & Verhulst 2023; Jalilpour Monesi et al. 2024), a psychometric curve for this test has been developed only from data related to TH listeners and is available only through an unpublished white paper (Luts et al. 2014). Psychometric curves for the DIN test and LIST sentences also have yet to be reported for CI users, so the steepness of the slopes associated with this population is unknown. Previous work leads us to expect the slopes to be lower than for TH listeners (for a review, see MacPherson & Akeroyd 2014).

For all three tests, comparative data between CI users and TH listeners are available for the SRT, but only some are based on age-matched populations, complicating comparisons among studies (Goossens et al. 2017). Generally, SRTs are substantially higher for CI users than for TH listeners. Claes et al. (2018) identified an average difference of approximately 5 to 6 dB. The SRTs for CI users show considerable variability due to the heterogeneity within this population. Factors that can influence SI in this group include the age at which they received their implant, the duration of deafness, and residual hearing (Blamey et al. 2013).

In this work, we compared psychometric curves associated with CI users to curves associated with age-matched TH listeners using three speech corpora (digit triplets, Matrix sentences, and sentences with lexical context). We also investigated the effect of the scoring method on the psychometric curves. Using the results, we made detailed comparisons of the psychometric slopes and SRTs with those obtained from the literature. This analysis included descriptive statistics of these outcome measures based on their estimated effect sizes, which capture the sensitivity and variance of the speech tests across groups and scoring methods.

On the basis of the literature, our overall prediction was that the slopes would be shallower and the SRTs higher (less favorable) for CI users compared with TH listeners. In both participant groups, we expected to see the steepest slopes for the LIST sentences and the DIN test. In our comparison of the effects of sentence scoring versus word scoring, we expected sentence scoring to yield an increased slope. Enhanced steepness would be especially beneficial for testing CI users, whose results typically yield shallow slopes and thus poor sensitivities of the correct score to changes in SNR. In assessing the feasibility of using word (digit) scoring for the DIN and LIST tests, we expected word scoring to be helpful, especially for the LIST sentences, as the available number of lists (35 with 10 sentences each) restricts its use in studies with testing under many conditions. By using word scoring instead of sentence scoring, potentially fewer items are needed to reach a given accuracy.

## MATERIALS AND METHODS

### Study Design and Participants

CI users were recruited by written invitation. We included 18 experienced, unilateral CI users (mean age ± SD: 63 ± 5 years) who were implanted with Advanced Bionics devices (Valencia, CA, USA) at the Leiden University Medical Center (see Table 1 in Supplement Digital Content, http://links.lww.com/EANDH/B659, for the demographics). No cases involving single-sided deafness were included, and contralateral residual hearing was minimal. Most participants did not use a hearing aid in their contralateral ear. To facilitate assessment of the effects of a CI on the psychometric curve, any assistive devices were removed from the contralateral ear, which was then plugged. Participants were selected based on a monosyllabic consonant-vowel-consonant phoneme score in quiet of 75% or higher (equivalent to a word score >50%) (Bosman & Smoorenburg 1987; Bosman et al. 1992), length of experience with their CI (>3 years), and age (adults ≤75 years). Care was taken to ensure that each individual was naïve to the speech materials. Participants were fitted with a research speech processor (Q90, Advanced Bionics LLC, Valencia, CA) using their threshold and maximal comfortable stimulus levels. To further standardize listening conditions across the participants and ascertain that SNRs were set as intended, any front-end processing strategies (e.g., noise reduction algorithms) were deactivated.

This group of CI users was compared with 18 age-matched TH individuals (62 ± 12 years) mainly recruited via flyers in our hospital and through referrals from the CI users, often their family members or friends. TH listeners did not use hearing devices in daily life, and their pure-tone audiometric thresholds were not worse than 30 dB at any frequency (125–8000 Hz) than the mean threshold in their age group according to the ISO 7029:2017-06 standard (Michel 2021; Kurakata 2023).

This research adhered to the tenets of Helsinki (World Medical Association 2013) and was approved by the local medical ethical committee (institutional review board) of Leiden, Den Haag, Delft (METC LDD) under study number P8.177. It was registered in the Dutch Trial Register of the Central Committee on Research Involving Human Subjects (CCMO) under trial number NL67179.058.18 (https://onderzoekmetmensen.nl/en/trial/52777) on October 3, 2022. All participants signed informed consent.

### Test Environment

Speech tests were performed in a sound-attenuated booth measuring 3.4 × 3.2 × 2.4 m (l × w × h). Speech and noise were presented using a calibrated (Rion NA-28, Rion Co. Ltd., Tokyo, Japan) loudspeaker (KEF, Ci100QS, GP Acoustics, Kent, UK) with a flat frequency response situated in front of the participant at ear level and at a distance of approximately 1 m.

### Speech Recognition Testing

Speech recognition was assessed using the DIN test (Smits et al. 2013), the Matrix test (Luts et al. 2014), and the LIST sentences (Van Wieringen & Wouters 2008). All materials were spoken in Dutch. The DIN test was developed in Dutch in the Netherlands, and the Matrix material and LIST sentences were developed in Flanders, the Dutch-speaking part of Belgium. Participants listened to the stimulus (a sentence or triplet in the case of the DIN test) and verbally repeated it to the experimenter. Guessing was allowed, but no feedback was provided during testing. Before the psychometric curve of a particular speech test was determined, two practice lists were first applied: one in quiet and one in noise (+6 dB SNR). In the case of the Matrix test, a sheet with the 50-word Matrix was available to the participant during the practice tests to reduce learning effects associated with this test (Stronks et al. 2022). In this work, the DIN and Matrix tests were scored based on verbal responses. We opted for verbal answers from our participants to ensure that the SpiN tests were all administered as similarly as possible, as verbal responses are the only feasible method for the LIST sentences. These tests are often scored with the items being available to the listener.

The corresponding long-term speech-shaped noise was used for each speech test and presented at a constant level of 60 dBA. Noise preceded and followed the target by 2 sec. Two seconds of noise is sufficient for the CI’s adaptive gain control to adjust to the appropriate level (Boyle et al. 2009). The room was quiet between stimulus presentations and during answering.

Each speech test features a corpus with different syntactic, lexical, and semantic content. Still, all were developed to adaptively determine the speech reception threshold (SRT), defined as the SNR where SI = 50%. Every test comes with an adaptive protocol, but here, we used the method of constant stimuli to determine the psychometric curve instead. SI was assessed with word (digit) and sentence (triplet) scoring.

The DIN test (Smits et al. 2013) consists of five lists of 24 triplets, which was extended to 10 lists by randomizing the order of the triplets within each original list. This test was designed for triplet scoring. The Matrix test consists of 13 lists of 20 sentences of semi-random combinations of five words drawn from a set of 50, so that each sentence consists of a name, verb, quantity, color, and object. It was designed as a word-scoring test (Luts et al. 2014). The LIST sentences (Van Wieringen & Wouters 2008) comprise 35 lists of 10 meaningful sentences of varying lengths and difficulty. It was originally developed for sentence scoring, so a sentence is considered correct only when all keywords are correct.

All words (digits) per sentence (triplet) were scored for the DIN and Matrix tests. For the LIST sentences, only the keywords were taken into account, as described by Van Wieringen and Wouters (2008). For the DIN test, digit scoring was performed in a place-specific manner, identical to the original triplet scoring instructions (Smits et al. 2013). To facilitate this method, participants always provided three responses per triplet, typically consisting of three digits but potentially including “I don’t know,” “no,” or any equivalent negative for a specific digit. With place-specific scoring, the answer “3 2 1” to a target triplet of “1 2 3” yields a correct digit score of 1 and a triplet score of 0. An answer “no 2 3” would yield a correct digit score of 2 and a triplet score of 0, whereas “3 1 2” would deliver a score of 0 for both methods, even though all digits in the answer were present in the target triplet. For the Matrix test and LIST sentences, words were scored regardless of their position in the sentence. The participants received explicit explanations of the scoring methods, but no feedback was given after the practice tests concluded.

Because we did not apply the SpiN tests adaptively, the original lists of the DIN and Matrix tests were divided in two, effectively doubling the available number of lists. The LIST material consisted of lists of 10 sentences and was not adapted for this study. Ten sentences (12 triplets) were sufficient to obtain a reliable percent correct score for the method of constant stimuli. SNRs and lists were randomized per participant. Speech tests were conducted using custom-built software and executed in a MATLAB R2021a programming environment (MathWorks, Inc., Natick, MA, USA). Test conditions were provided in a randomized block design, as follows: On the first day, one randomized speech test was provided, and on the second day, the remaining two were provided, also in random order. The list numbers and sequences of SNRs were randomized across participants.

### Construction of Psychometric Curves

Only word (digit) scores were assessed during the test session, and sentence scores were obtained and evaluated post hoc. Word (digit) scores were obtained at seven fixed SNRs, including the expected SRT and at SNRs 3, 6, and 9 dB above and below the expected SRT. Expected SRTs were drawn from the literature, with an emphasis on studies with participant groups in an age range comparable to ours. The SRTs obtained from the literature were based on word scoring for the Matrix test and sentence/triplet scoring for the other tests. For the DIN test, these values were −2 dB SNR for CI users (Kaandorp et al. 2015) and −12 dB SNR for TH listeners (Smits et al. 2013; Kaandorp et al. 2015; Dambha et al. 2022). For the Matrix test, the estimated SRT was −2 dB SNR for CI users (Langerak et al. 2024) and −4 dB SNR for TH listeners. The latter value was based on a reported SRT of −9 dB SNR in young TH listeners (Luts et al. 2014) and was increased by 5 dB SNR to account for older age (Van Wieringen & Wouters 2008; Claes et al. 2018). For the LIST sentences, expected SRTs were +8 dB SNR for CI users and −3 dB SNR for TH listeners (Claes et al. 2018).

In some cases, these predetermined SNRs needed adjustment to ensure sufficient data for reliable curve fitting. This adjustment was especially needed for TH listeners, whose results often yielded steep sloping curves around the SRT, so that additional data needed to be collected in this SNR range. Criteria for fitting a psychometric curve were based on word (digit) scores and were as follows: to ensure sufficient measurements around SRT, (1) at least one measurement was available between 10% and 50% correct score, and (2) at least one measurement was available between 50% and 90%, and (3) these previous data points were less than 40% apart. To ensure accurate estimation of the asymptotes, (4) at least one measurement was obtained near the upper asymptote (above ~90% correct), and (5) at least one measurement was obtained near the lower asymptote (~10%). Last, (6) there were at least seven scores at different SNRs available. When these criteria were not met, additional data points were collected at appropriate SNRs, depending on the part of the curve that needed improvement. In addition, when curve fits delivered unacceptable results, for instance, when most data appeared on either side of the curve or when the slope of the fitted curve was substantially higher than expected (i.e., a slope higher than ~35%·dB^−1^ or lower than ~5%·dB^−1^), additional points were collected around the expected SRT. These word/digit score–based criteria were sufficient to generate reliable sentence/triplet score–based curves for the DIN test and LIST sentences. However, sentence scores for the Matrix test were often worse than their corresponding word scores, which sometimes resulted in an underrepresentation of data points around the upper asymptote *λ*.

The data were fitted with an inverse log-Weibull (Gumbel) function (Gilchrist et al. 2005; Zychaluk & Foster 2009; Kingdom & Prins 2010):


$$\begin{array}{l} Y=\gamma +\left(1-\gamma -\lambda \right)\cdot e^{-10^{\beta \left(\alpha -X\right)}} \end{array}$$ (1)

where Y is the correct score (fraction), γ is the guess rate (fraction), λ is the lapse rate (fraction), β is a slope parameter, α is the threshold (speech level in dBA), and x is speech level (dBA). Hereafter, we express speech levels in dB SNR (re. 60 dBA of long-term speech-shaped noise) and correct rates in percentages for clarity. In Eqs. (1) and (2), however, fractions and speech levels were used as input, respectively.

**Fig. 1.**
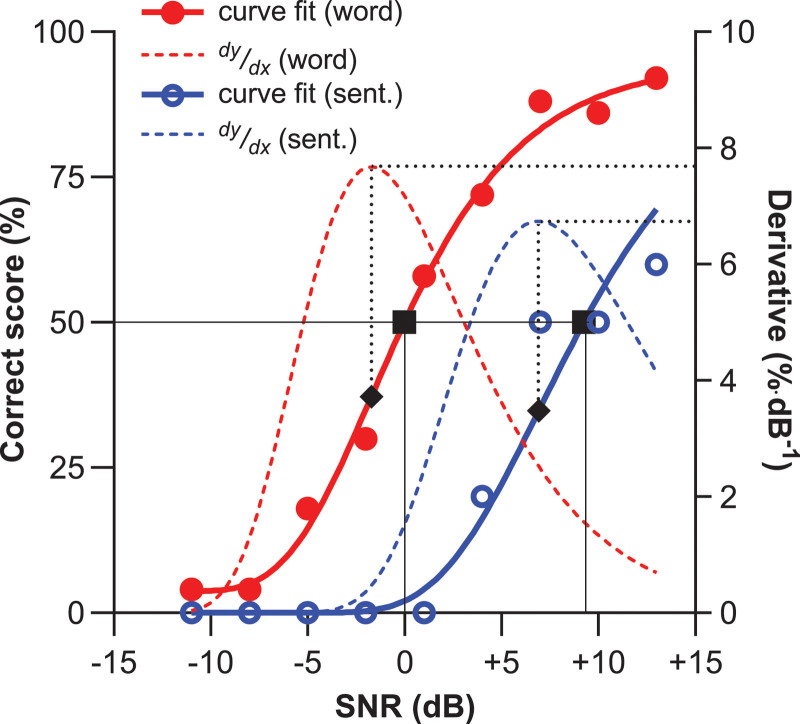
Sample fits of psychometric curves of a CI user performing the Matrix test. Word scores (closed red circles) and sentence scores (sent. open blue circles) were fitted with a reversed Gumbel function (solid red and blue lines). The first derivatives reflect the slope of the curve as a function of SNR (dotted red and blue lines). The inflection points of the derivatives correspond to the steepest point on the psychometric curve (dotted black lines). This point corresponds to the curve’s threshold *α* (black diamonds). SRTs (black squares) correspond to the SNR where SI was 50% (black solid lines). Because the Gumbel function is asymmetric, *α* deviates from the SRT and invariably occurs at slightly lower SNRs. CI indicates cochlear implant; SI, speech intelligibility; SNR, signal to noise ratio; SRT, speech reception threshold.


The Gumbel function is asymmetric and used for logarithmic stimulus levels (Kingdom & Prins 2010). The reverse Gumbel function fitted our data well because the bottom part of this function is somewhat steeper sloping than the top (Fig. 1). Fitting was performed with a maximum likelihood procedure using the Palamedes Toolbox version 1.11.11 for MATLAB (Prins & Kingdom 2018). Extracted parameters from the curve fits were β, SRT, γ, and λ. Sample curve fits using word and sentence scoring for a CI user performing the Matrix test are shown in Figure 1. In contrast with symmetric functions, such as the often-used logistic fit (MacPherson & Akeroyd 2014), the SNR at the steepest part of the reverse Gumbel function *α* (black diamonds in Fig. 1) did not precisely match the slope at SRT (black squares in Fig. 1), so that the steepest slope of the curve did not match the slope at SRT. *α* always was located lower on the curve because of the characteristics of the reverse Gumbel function, but SRT and *α* were consistently located on the near-linear part of the curve, and the slopes at *α* and SRT did not differ substantially. Here, we report the SRT (i.e., the SNR at 50% words correct) and the slope at *α* (i.e., the steepest slope of the curve). The first derivatives are shown for illustrative purposes in Figure 1 and reflect the slope of the two fits as a function of SNR corresponding to:

$$\begin{array}{l} \frac{dY}{dX}=\ln\left(10\right)\cdot \beta \cdot \left(1-\gamma -\lambda \right)\cdot 10^{\beta \left(\alpha -X\right)}\cdot e^{-10^{\beta \left(\alpha -X\right)}}) \end{array}$$ (2)

where $\frac{dY}{dX}$ is the slope of the psychometric function (in fraction·dB^−1^).

### Statistical Analysis

To compare slopes and SRTs across speech tests and groups, statistical significance testing was performed by constructing linear mixed models (LMMs) fitted with the restricted maximum likelihood procedure using IBM SPSS Statistics for Windows 29.0 (released 2022, IBM Corp. Armonk, NY). Speech tests (DIN, Matrix, or LIST), group (TH or CI), and their interaction were included as fixed factors. Participant ID was used as a random variable, according to:


y~SpeechTest+TH/CI+(SpeechTest*TH/CI)+1|participant (3)

where y is the dependent variable (slope, SRT), SpeechTest is a categorical repeated measures variable for the type of speech tests (DIN, Matrix, or LIST), TH/CI is a categorical variable for the TH or the CI group, and participant is a random intercept factor. In the equation, * indicates an interaction factor.

When an LMM delivered significant effects, post hoc multiple comparisons significance testing was performed on the estimated marginal (EM) means between pairs of conditions using Šidák’s correction (Abdi 2007). All possible post hoc combinations were tested when the interaction term was significant. In this case, significance testing was performed between pairs of speech tests within each group and between the two participant groups for each of the speech tests. In case the interaction term was not significant, the effect of the speech test was assessed post hoc by significance testing of the EM mean across groups, and the effect of the group was evaluated by significance testing of the EM mean across speech tests.

The covariance matrix of the repeated variable (speech test) was determined for each LMM by comparing nine often-used structures for longitudinal data (“scaled identity,” “compound symmetry” and its “correlation”; and “heterogeneous counterparts,” “unstructured” and its “correlations” counterpart; and “Toeplitz” and its “heterogeneous counterpart”). Comparisons between the models were based on the Bayesian information criterion, which is recommended for small samples (Gurka 2006), using restricted maximum likelihood, also as recommended (Wolfinger 1993). The covariance structure of the random variable (i.e., participant) was always set at the default “scaled identity” because only a single random variable was present. Degrees of freedom were determined using the method of Satterthwaite (1946). The remaining parameters were left at default per SPSS v. 29.0.

For comparison of the sensitivities of the three speech tests for detecting changes in SI in the two groups, the effect sizes were calculated as Cohen *d*_*z*_ (Lakens 2013):


$$\begin{array}{l} d_{z}^{}=\frac{M_{dif\;f}^{}}{S_{dif\;f}^{}} \end{array}$$ (4)

where $d_{z}^{}$ is the effect size in a paired samples *t* test (or the equivalent one-sample test of the differences), $M_{dif\;f}^{}$ is the mean difference, and $S_{dif\;f}^{}$ is the SD of the difference. For the slope, $M_{dif\;f}^{}$ equaled the effect of a 1 dB shift in SNR on the percent correct score. $M_{dif\;f}^{}$ thus depended on the slope and the variance. For the SRT, $M_{dif\;f}^{}$ was set at 1 dB SNR, and $d_{z}^{}$ thus reflected the effect size when SRTs differed by 1 dB and depended only on the variance of the SRT. A $d_{z}^{}$ of 0.8 can roughly be interpreted as a large effect, 0.5 as medium, and 0.2 as small (Cohen 1988). Alternatively, these cutoffs can be seen as indicating obvious, subtle, and merely statistical effects (Fritz et al. 2012). The $d_{z}^{}$, its input values, and the descriptive statistics for the different speech tests and the two groups can be found in Tables 1–4. Tables 1–6 are based on the raw data and serve as an accessible reference for the population means. However, the main text includes EM means or EM mean differences. These EM means were derived from LMM outcomes and were more suitable for statistical post hoc (multiple comparisons) testing. EM means are more robust against potential confounders, which are included as fixed and random factors in the LMM. However, we encourage using the “simple” means in the tables for descriptive purposes and to aid future researchers in reproducing our results.

**TABLE 1.** Descriptive statistics for slopes using word/digit scoring (N = 18)

Group	Test	Mean (%·dB^−1^)	SD (%·dB^−1^)	95% CI (%·dB^−1^)	Cohen *d*_*z*_
CI	Matrix	7.7	2.2	6.6–8.8	3.5
	LIST	12.5	6.3	9.3–15.6	2.0
	DIN	12.5	2.8	11.1–14.0	4.4
TH	Matrix	17.3	4.0	15.3–19.3	4.3
	LIST	18.5	3.4	16.8–20.2	5.4
	DIN	20.8	5.0	18.3–23.3	4.1

CI, cochlear implant; Cohen *d*_*z*_, effect size in a paired-samples *t* test when the performance difference equals the mean slope, that is, the performance difference at an SNR change of 1 dB; TH, typical hearing.

**TABLE 2.** Descriptive statistics for SRT using word/digit scoring (N = 18)

Group	Test	Mean (dB)	SD (dB)	95% CI (dB)	Cohen *d*_*z*_
CI	Matrix	0.9	2.7	−0.4 to 2.3	0.4
	LIST	0.2	2.9	−1.3 to 1.6	0.3
	DIN	−5.2	1.5	−4.5 to −5.9	0.7
TH	Matrix	−7.6	1.2	−7.0 to −8.2	0.8
	LIST	−8.3	1.1	−8.0 to −8.9	0.9
	DIN	−10.2	0.8	−9.8 to −10.6	1.2

CI, cochlear implant; Cohen *d*_*z*_, effect size in a paired samples *t* test when the SRT difference equals 1 dB SNR; TH, typical hearing.

**TABLE 3.** Descriptive statistics for slope using sentence/triplet scoring

Group	Test	Mean (%·dB^−1^)	SD (%·dB^−1^)	95% CI (%·dB^−1^)	N*	Cohen *d*_*z*_
CI	Matrix	5.9	2.1	4.8–7.0	17	2.8
	LIST	10.6	3.2	8.8–12.3	16	3.3
	DIN	15.6	7.4	11.9–19.2	18	2.1
TH	Matrix	15.7	4.7	13.1–18.3	15	3.4
	LIST	19.7	6.3	16.6–22.8	18	3.1
	DIN	24.4	8.0	20.3–28.5	17	3.0

* Some values missing because of unreliable curve fits resulting from a lack of data.

CI, cochlear implant; Cohen *d*_*z*_, effect size in a paired-samples *t* test when the performance difference equals the mean slope, that is, the performance difference at an SNR change of 1 dB; TH, typical hearing.

**TABLE 4.** Descriptive statistics for SRT using sentence/triplet scoring*

Group	Test	Mean (dB)	SD (dB)	95% CI (dB)	Cohen *d*_*z*_
CI	Matrix	10.4	6.5	7.1 to 13.7	0.2
	LIST	2.2	3.1	0.7 to 3.7	0.3
	DIN	−1.5	2.5	−2.7 to −0.2	0.4
TH	Matrix	−4.0	1.6	−4.8 to −3.2	0.6
	LIST	−6.9	1.3	−7.5 to −6.2	0.8
	DIN	−8.4	0.6	−8.7 to −8.1	1.7

* N = 18, except Matrix for CI, where N = 17 due to a non-converging curve-fit because of a lack of data around the SRT.

CI, cochlear implant; Cohen *d*_*z*_, effect size in a paired samples *t* test when the SRT difference equals 1 dB SNR; TH, typical hearing.

**TABLE 5.** Comparison of slopes with those reported in the literature

Test	CI	TH		
	Slope ± SD (N)	Slope ± SD (N)	Ref. Slope ± SD (N)	Source
Matrix	7.7 ± 2.2 (18)	17.3 ± 4.0* (18)	13.9 ± 1.5 (20)	Luts et al. (2014)†
LIST	10.6 ± 3.2 (16)	19.7 ± 6.3 (17)	17.5 ± 2.0 (10)	Van Wieringen and Wouters (2008)†
DIN	15.6 ± 7.4 (18)	24.4 ± 8.0* (17)	18.4 ± 1.5 (9)	Smits et al. (2016)†

Reference values for the slopes in CI are unavailable.

* Current data significantly different from literature (*p* < 0.01).

† Slopes from young TH listeners. Data were obtained with word scoring for the Matrix test and sentence and triplet scoring for the LIST and DIN tests, respectively.

CI, cochlear implant; TH, typical hearing.

**TABLE 6.** Comparison of curve-fit–based SRTs from this study with adaptive SRTs obtained from the literature

Test	CI			TH		
	SRT ± SD (N) This Study	Adaptive SRT ± SD (N) Literature	Source	SRT ± SD (N) This Study	Adaptive SRT ± SD (N) Literature	Source
Matrix	0.9 ± 2.7 (18)	−3.6 ± 2.4** (17)	Stronks et al. (2021)†‡	−7.6 ± 1.2 (18)	−9.5 ± 0.8** (20)	Luts et al. (2014)§
LIST	2.2 ± 3.1 (18)	8.1 ± 7.1** (61)	Claes et al. (2018)†¶	−6.9 ± 1.3 (18)	−3.1 ± 2.5** (81)	Claes et al. (2018)§
DIN	−1.5 ± 2.5 (18)	−1.8 ± 2.7 (24)	Kaandorp et al. (2015)†	−8.4 ± 0.6 (18)	−8.8 ± 0.6* (23)	Smits et al. (2013)§∥

Data were obtained with word scoring for the Matrix test and sentence and triplet scoring for the LIST sentences and DIN test, respectively.

† SRTs obtained in an age group approximating ours.

‡ SD not reported in Stronks et al. (2021) and calculated from the original data set; some participants had substantial contralateral residual hearing, and most had experience with the Matrix test.

§ SRTs from young TH listeners.

¶ Study population included some bimodal listeners.

∥ Median SRT in an age group similar to this study was −7.4 dB (Table 3 in Smits et al. 2013).

* *p* < 0.05,

** *p* < 0.001 compared with data reported in the literature.

CI, cochlear implant; SRT, speech recognition threshold; TH, typical hearing.

Lapse and guess rates were not statistically assessed. The median 1 − *γ* was 95% or higher across speech tests and both participant groups for word/digit scoring and higher than 90% for sentence/triplet scoring. Median guess rates were 0% across tests, participant groups, and scoring methods.

## RESULTS

### Fitting of Psychometric Curves

**Fig. 2.**
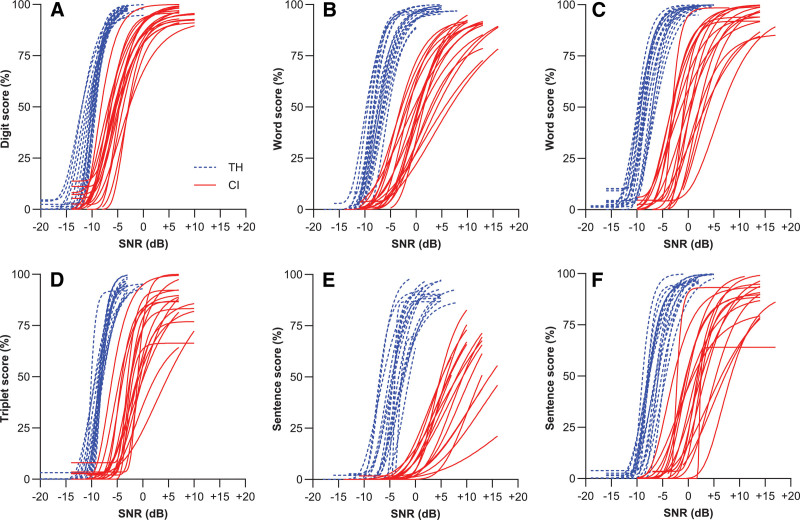
Fitted psychometric curves. Curves based on word scoring (A–C) and sentence scoring (D–F) are shown for CI users (solid red curves) and TH listeners (dashed blue curves) for the DIN test (left panels), Matrix test (middle), and LIST sentences (right). CI indicates cochlear implant; TH, typical hearing.

The individual psychometric curve fits for the three speech tests, two groups, and the two scoring methods are shown in Figure 2. The descriptive statistics of the slopes and SRTs extracted from these curves are provided in Tables 1 and 2 (word/digit scoring) and Tables 3 and 4 (sentence/triplet scoring). For CI users, curves consistently had shallower slopes (red lines in Fig. 2) and higher SRTs compared with TH listeners (blue lines). The most profound effect of scoring method was observed for the Matrix test; compared with word scoring (Fig. 2B), sentence scoring (Fig. 2E) unexpectedly and markedly reduced the slope of the psychometric curves and deteriorated SRTs and overall scores, especially for CI users. For the DIN test (Figs. 2A, D), the curves for the CI users were shifted substantially to the right when triplet scoring was used, indicating higher SRTs in this group. In both groups, the score method had little effect on the slopes. For the LIST sentences (Fig. 2C, F), slopes increased somewhat but with a negligible effect on SRTs.

### Slopes and SRTs Using Word and Digit Scoring

**Fig. 3.**
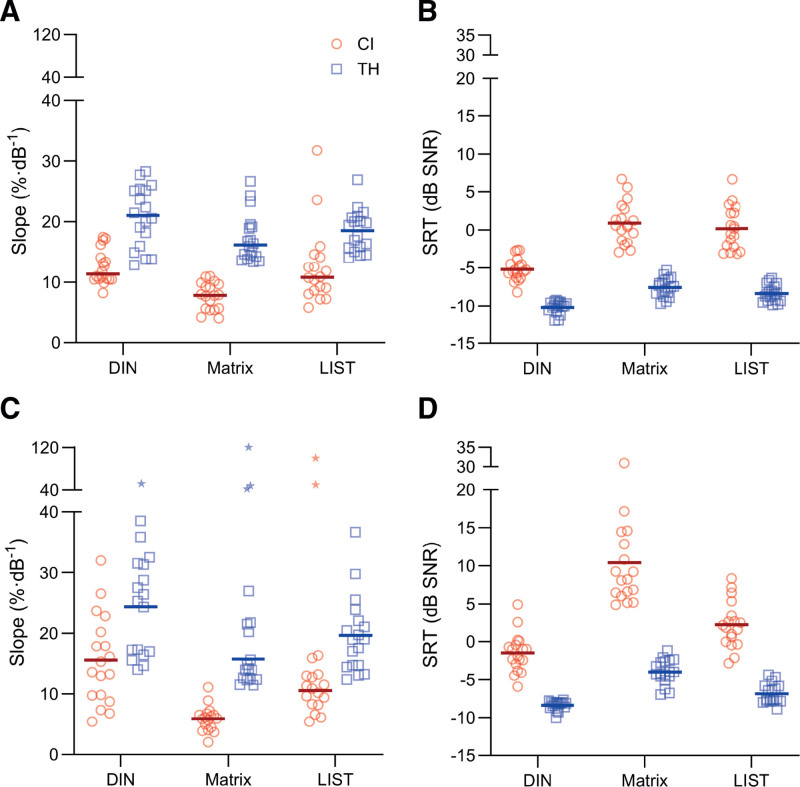
Outcome measures extracted from the psychometric curves. Slopes and SRTs for word scoring (A, B resp.) and sentence scoring (C, D) are shown for CI users (red circles) and TH listeners (blue squares). On the basis of Tukey method, slopes obtained with sentence scoring (C) resulted in outliers (stars). Solid lines: means. CI indicates cochlear implant; SRT, speech reception threshold; TH, typical hearing.

Scatter plots of the slopes and SRTs extracted from the psychometric curves using word/digit scoring are shown in Figs. 3A, B, respectively. For the slope, an LMM with a diagonal covariance matrix for the repeated measured variable (speech test) was used to assess statistical differences between speech tests and groups. This LMM revealed a significant main effect of speech test [*F*(2,68) = 9.9, *p* < 0.001] and group [*F*(1,34) = 87.0, *p* < 0.001], but not of the interaction term [*F*(2,68) = 1.7, *p* = 0.184].

On the basis of the absence of interaction, post hoc significance testing of the effect of speech test on the slope was performed by comparing the tests across groups (i.e., after “pooling” TH and CI), and the TH and CI groups were compared across (pooled) speech tests. The EM mean slope (%·dB^−1^) for the DIN test was 16.7 (95% confidence interval [95% CI]: 15.3 to 18.1); for the Matrix test, it was 12.5 (11.1 to 13.9); and for the LIST sentences, it was 15.5 (14.1 to 16.9). The SE was 0.7%·dB^−1^, and degrees of freedom (df) was 101 for these three EM mean slopes. The differences (with 95% CI) among EM mean slopes (%·dB^−1^) across groups were significant between the DIN and the Matrix test (4.2, 1.8 to 6.5, *p* < 0.001) and between the Matrix test and LIST sentences (3.0, 0.6 to 5.3, *p* = 0.009), but not for the DIN test and LIST sentences (1.2, −1.2 to 3.6, *p* = 0.519). SE was 1.0%·dB^−1^, and df was 68 for these three comparisons.

The EM mean slope averaged across speech tests was 10.9%·dB^−1^ (95% CI: 9.7 to 12.1) for the CI group and 18.9%·dB^−1^ (17.6 to 20.1) for the TH listeners, with SE = 0.6%·dB^−1^ and df = 34. The EM mean difference of 7.9%·dB^−1^ between the groups was statistically significant (SE: 0.9; 95% CI: 6.2 to 9.7; df: 34; *p* < 0.001).

For the SRT obtained with word/digit scoring, an LMM was constructed with a heterogeneous compound symmetry covariance matrix. The main effects of speech test [*F*(2,34) = 126, *p* < 0.001], group [*F*(1,34) = 177, *p* < 0.001], and interaction term [*F*(2,34) = 21.7, *p* < 0.001] were significant. On the basis of the significant interaction term, the differences between speech tests were compared post hoc within each group, and the between-group effect was investigated per speech test.

The EM mean SRT (dB SNR) for the CI group was −5.2 for the DIN test (SE: 0.3; 95% CI: −5.8 to −4.6; df: 34), 0.9 for the Matrix test (0.5, −0.9 to 1.9, 36), and 0.2 for the LIST sentences (0.5, −0.9 to 1.2, 36). The SRT for the TH listeners was −10.2 for the DIN test (SE: 0.3; 95% CI: −10.8 to −9.6; df: 34), −7.6 for the Matrix test (0.5, −8.6 to −6.6, 36), and −8.3 for the LIST sentences (0.5, −9.4 to −7.3, 36).

Post hoc testing revealed a significant difference (dB SNR) in the SRTs between every pair of speech tests in both groups. In the CI group, SRTs differed by 6.1 (SE: 0.4; 95% CI: 5.1 to 7.1; *p* < 0.001) for the DIN and Matrix tests, by 5.4 (0.4, 4.3 to 6.4, *p* < 0.001) for the DIN test and LIST sentences, and by 0.8 (0.3, 0.0 to 1.5, *p* = 0.049) for the Matrix test and LIST sentences. In the TH group, SRTs differed by 2.6 (0.4, 1.7 to 3.6, *p* < 0.001) for the DIN and Matrix test, by 1.9 (0.4, 0.8 to 2.9, *p* < 0.001) for the DIN test and LIST sentences, and by 0.8 (0.3, 0.0 to 1.5, *p* = 0.043) for the Matrix test and LIST sentences. The df value among the comparisons was 34.

As expected, CI users had significantly higher SRTs than TH listeners. For the DIN test, the EM mean difference between groups was 5.0 dB SNR (SE: 0.4; 95% CI: 4.2 to 5.8; df: 34). For both the Matrix test and the LIST sentences, this difference was 8.5 dB SNR (Matrix: 0.7, 7.1 to 9.9, 36, *p* < 0.001; LIST: 0.7, 7.0 to 10.0, 36, *p* < 0.001).

### Slopes and SRTs Using Sentence and Triplet Scoring

The data obtained for the slopes and SRTs using sentence/triplet scoring are shown in Figures 3C, D, respectively. These data were more variable than those obtained with word/digit scoring, at least in part because the criteria used to optimize the curve fits were applied only for word/digit scoring. As a result, the psychometric curves obtained with sentence/triplet scoring typically had fewer data available around SRT, and the upper asymptote (1 − *λ*) was often undersampled. Some of these undersampled curves resulted in unrealistically steep slopes. Outliers were identified using Tukey’s criterion of 1.5 times the interquartile range (Páez & Boisjoly 2022). Slope estimates outside this range were removed from the analysis (asterisks in Fig. 3C) and from the descriptives in Table 3. LMMs are well suited for analyzing data sets with missing data. SRT estimates from the curve fits with excluded slopes were considered reliable enough to include in the analyses.

The LMM of the slopes obtained with sentence scoring was constructed with a diagonal covariance matrix. The result revealed a significant main effect of speech test [*F*(2,31) = 37.2, *p* < 0.001] and group [*F*(1,45) = 41.1, *p* < 0.001], but not of the interaction term [*F*(2,31) = 0.1, *p* = 0.883], similar to the outcomes with word/digit scoring. The EM mean of the slope (%·dB^−1^) across both groups was 20.3 (SE: 1.5; 95% CI: 17.2 to 23.4; df: 35) for the DIN test, 10.7 (0.6, 9.5 to 11.9, 33) for the Matrix test, and 15.1 (0.8, 13.5 to 16.7, 43) for the LIST sentences. The EM mean differences (%·dB^−1^) between the slopes were significant for every pair of speech tests. The difference was 9.6 (SE: 1.5; 95% CI: 5.9 to 13.3; df: 34; *p* < 0.001) for the DIN and Matrix tests, 5.2 (1.6, 1.3 to 9.0, 40, *p* = 0.006) for the DIN test and LIST sentences, and 4.4 (0.7, 2.6 to 6.2, 26, *p* < 0.001) for the Matrix test and LIST sentences. The EM mean slope, averaged across speech tests, was 10.6 for the CI group (SE: 1.0; 95% CI: 8.5 to 12.7; df: 45) and 20.1 for the TH listeners (1.1, 18.0 to 22.246). The resulting difference of 9.5 was statistically significant (1.5, 6.5 to 12.5, 45, *p* < 0.001).

For the SRT, the LMM was constructed with a Toeplitz covariance matrix. It revealed a significant main effect of speech test [*F*(2,44) = 91.5, *p* < 0.001], group [*F*(1,30) = 114, *p* < 0.001], and the interaction term [*F*(2,44) = 20.9, *p* < 0.001]. On the basis of the significant interaction term, we compared the differences between speech tests in each group and investigated the between-group effect per speech test. The EM mean SRT (dB SNR) for the CI group was −1.5 for the DIN test (95% CI: −3.1 to −0.1), 10.6 for the Matrix test (9.0 to 12.2), and 2.2 for the LIST sentences (0.6 to 3.8). The SRT for TH listeners was −8.4 (−10.0 to −6.8) for the DIN test, −4.0 (−5.6 to −2.4) for the Matrix test, and −6.9 (−8.5 to −5.3) for the LIST sentences. For all EM means, the SE was 0.8 and the df was 52.

Within both the CI and TH groups, SRTs differed significantly between each pair of speech tests, except for SRTs obtained with the LIST sentences and the DIN test in TH listeners. In the CI group, the EM mean SRT differed by 12.1 (SE: 0.99; 95% CI: 9.8 to 14.4; df: 54, *p* < 0.001) for the DIN and Matrix tests, by 3.7 (0.6, 2.1 to 5.2, 63, *p* < 0.001) for the DIN test and LIST sentences, and by 8.4 (0.6, 6.8 to 10.0, 64, *p* < 0.001) for the Matrix test and LIST sentences. In the TH group, the SRTs for the DIN and Matrix test differed by 4.4 (0.9, 2.1 to 6.7, 53, *p* < 0.001), and those for the Matrix and LIST differed by 2.9 (0.6, 1.3 to 4.4, 63, *p* < 0.001). A difference of 1.5 did not reach significance for the LIST sentences and DIN test (0.6, 0.0 to 3.1, 63, *p* = 0.06). Similar to the results obtained with word/digit scoring, the CI users had significantly higher SRTs than the TH listeners. The EM mean difference (dB SNR) between the groups was 6.9 (95% CI: 4.7 to 9.2; df: 52; *p* < 0.001) for the DIN test, 14.6 (12.3 to 17.0, 53, *p* < 0.001) for the Matrix test, and 9.1 (6.8 to 11.3, 52, *p* < 0.001) for the LIST sentences. For the three comparisons, the SE was 1.1.

### Comparison of Effect Size

The effect sizes, expressed as Cohen *d*_*z*_, are included in Tables 1–4 and are convenient for comparisons between speech tests because *d*_*z*_ captures the effect magnitude and variance in a single measure (Eq. 4). The *d*_*z*_ values are based on the means and SDs of the raw data. The slope of the psychometric curve represents the sensitivity of a test in detecting changes in SNR via correct scores. The psychometric slope for the Matrix test was the shallowest for both word and sentence scoring. Slopes for the LIST sentences and DIN test were relatively steep, but because of the lower variance of the Matrix test, the *d*_*z*_ of the slopes was similar among the tests. The *d*_*z*_ value for the Matrix test was better than the *d*_*z*_ for LIST sentences with word scoring (Table 1) and better than the *d*_*z*_ for the DIN test with sentence/triplet scoring in the CI group (Table 3). For the TH listeners, *d*_*z*_ was similar among tests, except that the LIST sentences had a markedly larger *d*_*z*_ than the other tests with word scoring.

The *d*_*z*_ of the SRT was determined for a hypothetical difference of 1 dB SNR so that *d*_*z*_ differences depended only on S_diff_ (Eq. 4). For both word/digit and sentence/triplet scoring, the SRT of the DIN test emerged with the largest effect size (lowest *S*_diff_) in both groups, whereas Matrix and LIST performed similarly.

## DISCUSSION

In this study, we compared the psychometric curves of three Dutch speech tests (DIN, Matrix, and LIST) using word/digit and sentence/triplet scoring for a group of CI users and age-matched TH listeners. As expected, the psychometric curves for CI users were shallower than those obtained for TH listeners (MacPherson & Akeroyd 2014), with slopes approximately half as steep as those of TH listeners. The slopes differed across speech tests, but no significant group interaction was observed for word/digit scoring or sentence/triplet scoring. The slope of the psychometric curve of the Matrix test was significantly shallower than those of the other two tests. By contrast, the slopes obtained with the LIST sentences and DIN test were similar and did not differ significantly for either word or sentence scoring.

### Implications for CI Users of a Shallow Psychometric Slope and High SRTs

SpiN tests are typically developed and benchmarked using a group of young TH listeners. The observed differences in the slopes of the psychometric curves between the two groups in this study underscore the need for the collection of normative data specifically for CI users. In addition, adaptive speech tests are generally optimized for TH listeners. This optimization includes the magnitude of the step size, which is the SNR change applied between trials to adjust the SNR level in the adaptive procedure. Consequently, step-size magnitudes optimized for TH listeners may be suboptimal for CI users, potentially influencing the effectiveness of convergence on the SRT.

As expected, SRTs were significantly and substantially higher for CI users than for the TH group (Kaandorp et al. 2015; Stronks et al. 2025). SRTs depended on the speech test used, and unlike the slopes, showed a significant interaction between the speech test and the group. Overall, the Matrix test produced the highest SRTs and DIN the lowest. The CI group demonstrated the largest difference between SRTs across speech tests, with a Matrix test EM mean SRT that was 6 dB SNR higher than that of the DIN test when word scoring was used, a difference that grew to 12 dB SNR with sentence scoring. These findings confirm earlier reports that the Matrix test is more challenging than digit or everyday sentence tests (Jansen et al. 2012; Reynard et al. 2022; Stronks et al. 2024). A plausible reason is the relatively high rate of speech of the Matrix sentences of ~2.5 words/sec. By comparison, the DIN triplets are uttered at a rate of ~1.5 digits/sec, while the LIST sentences have a reported rate of 2.5 syllables/sec (Van Wieringen & Wouters 2008), or ~1.7 words/sec assuming an average of 1.5 syllables per word (Yaruss 2000). In addition, the DIN test is linguistically and cognitively less complex than the other tests because of the small number of items per stimulus and the high likelihood of the items (Kaandorp et al. 2017). In contrast with the Matrix and DIN materials, the LIST sentences provide lexical context. Context allows for postdiction and thus provides additional cues, which is particularly important for CI users, who make substantially more use of context than TH listeners (Dingemanse & Goedegebure 2019). Taking these findings together, we conclude that the low lexical context, relatively high speech rate, and complexity of the sentences can make Matrix sentences challenging, especially for CI users.

### Implications of the Scoring Method

Adding items to digit stimuli increases the slope (Smits & Houtgast 2006). Triplet scoring thus should yield steeper curves than digit scoring, which is essentially equal to the average score across the three individual digits in a given triplet. Our findings are in agreement with these expectations, showing steeper slopes with triplet scoring for both groups (Tables 1 and 3). Against expectations, however, the Matrix test produced shallower slopes for sentence scoring than for word scoring in both participant groups. Overall, the differences between slopes obtained with the two scoring methods were confined to a low percentage per decibel, and we did not pursue statistical significance testing. Doing so would have added an extra factor and three additional interaction terms to the model, overly complicating interpretation of the LMMs. We conclude that word (digit) scoring yields comparable steepness of the psychometric functions and may be an attractive alternative for the DIN test and LIST sentences, given that triplet and sentence scoring, respectively, are the standards for these tests.

Sentence/triplet scoring invariably yielded higher SRTs than word/digit scoring, regardless of the speech test or participant group under consideration. In this case, again, however, we did not pursue further statistical testing because the effect was confined to no more than a few decibels for SNR (Tables 2 and 4). One exception to this pattern was the average SRT for the Matrix test for the CI group, which was substantially (almost 10 dB SNR) higher for sentence scoring. We conclude that sentence scoring may not be feasible for the Matrix test when testing CI users because it appears to be a challenging test for this population (Stronks et al. 2025). Consequently, we recommend administering this test with the usual word scoring for CI users.

### Comparison of Slope and SRT Estimates With the Literature and Implications of the Test Paradigm

Tables 5 and 6 compare the slopes and SRTs reported here to previously published findings, including statistical significance testing with unpaired *t* tests and Welch’s correction for unequal SDs. The slopes for TH listeners were significantly higher in our study compared with published results for the DIN and Matrix tests (see references listed in Table 5, *p* < 0.01). In the previous studies, the authors used a multiple-choice answer format for both speech tests, which is the method most commonly used for closed-set tests (Jansen et al. 2013; Kollmeier et al. 2015), and all possible items are available to the participant. Like all open-set tests, however, the LIST sentences can be administered only with an open-ended answer format, namely through verbal feedback, without visual information about the possible items in the sentence. We administered all three tests using an open-ended answer format to maintain similar testing conditions among the speech tests. The Matrix of words was available only during the Matrix test practice tests, and the integers used for the DIN test (1 to 9) were not shown at all. Participants did receive a detailed explanation of the nature of the test stimuli. Multiple-choice testing is generally associated with lower (i.e., “better”) SRTs, increased guess rates, different language processing, and fewer learning effects (Billings et al. 2023). We expect, however, that the method of scoring used here had little impact on the SRT because the reported effects are less than 1 dB SNR, at least for the Matrix test (Kollmeier et al. 2015). In the case of the DIN test, a forced choice paradigm is often used, requiring the listener to provide three digits before the subsequent item is played (Smits et al. 2013). In the current experiments, however, participants could leave blanks where needed. This deviation from the standard protocol might have affected the shape of the curve because guess rates would have been smaller in our paradigm. The Flemish accent also may have played a role in the Matrix test outcomes, given that the reference study included native speakers from Flanders (Van Wieringen & Wouters 2008), whereas we included Dutch participants. Other differences, such as age, also may have affected the results (Goossens et al. 2017).

Our reported SRTs obtained with the DIN test closely match those from the literature (see references in Table 6). SRTs obtained with the Matrix test were somewhat higher (worse) for both populations, while those for the LIST sentences were slightly lower (more favorable) than previously reported. The most obvious reason for these differences is that the reference SRTs were obtained with the use of adaptive procedures, whereas we adopted the method of constant stimuli to determine the psychometric relationships, as has been recommended for this purpose (Kaernbach 2001). These procedural differences may have led to deviations. In addition, population effects (as listed in Table 6) may have played a role. For instance, for the Matrix test the reference CI group in Table 6 was experienced with the test and used a contralateral hearing aid during testing. Both factors can potentially increase performance and thus explain the higher SRTs found in the present study (Stronks et al. 2022). Regarding the low SRTs found for CI users for the LIST sentences, another study found a median SRT of 0.7 dB SNR (Van Wieringen et al. 2021). This value is ~7 dB SNR lower than the reference paper used in Table 6 (Claes et al. 2018) and much closer to ours. The age range of the populations in these two reference studies was similar to ours, underscoring the heterogeneity in the CI population (Ertmer & Goffman 2011). Van Wieringen et al (2021) included a population with relatively good residual hearing which can have favored better SRTs (Blamey et al. 2013). compared with the reference study (Luts et al. 2014), we report relatively high SRTs for the Matrix test for TH listeners, which can be explained by the much older study population in the present study. The relatively favorable SRTs we find for TH listeners with the LIST sentences are not easily explained by population differences. We suspect that the adaptive test procedure used in the reference study may have been the cause of the discrepancy.

### Variance and Effect Size Measures of the Slope and SRT

The steepness of the psychometric curve of a speech test determines its sensitivity to SNR and its ability to reliably produce the SRT in an adaptive procedure (Visentin & Prodi 2018). The slope variance in a population also affects the reliability of a given speech test. We captured both these parameters by determining the effect size of the slope using Cohen *d*_*z*_. The shallowest slopes were observed for the Matrix test in the CI group for both word and sentence scoring. Nevertheless, because of the limited variance, the *d*_*z*_ associated with the slope from the Matrix test was better than those associated with LIST for word scoring and DIN for triplet scoring in the CI group. When comparing the *d*_*z*_ of the slopes between pairs of speech tests between the two groups (e.g., *d*_*z*_ of the Matrix test in the CI group versus *d*_*z*_ of the Matrix test in the TH group), we found little difference. One exception was the LIST sentences with word scoring, which had a *d*_*z*_ value for TH listeners that was more than twice that for CI users. The *d*_*z*_ of the slopes differed little between speech tests within the TH group but were more considerable in the CI group, particularly the *d*_*z*_ for digit scoring of the DIN test, which was twice that of the LIST sentences using word scoring. None of the tests stood out uniformly as the most sensitive test because the most favorable *d*_*z*_ of the slopes depended on both the type of scoring and the group under investigation.

The *d*_*z*_ for SRT differences depended only on the SD, and a low SD invariably yielded a high *d*_*z*_. The DIN test yielded the most favorable *d*_*z*_ among the speech tests for both types of scoring, while *d*_*z*_ values were similar between the other two tests. In the comparison of pairs of speech tests between the two groups, *d*_*z*_ was invariably better in the TH listeners. The *d*_*z*_ value increased substantially for the DIN test when triplet scoring was used, but the other tests revealed similar *d*_*z*_ values between both scoring methods. The speech test with the most favorable *d*_*z*_ for the SRT was the DIN test. For CI users, digit scoring appeared to be the most robust, whereas triplet scoring yielded more favorable effect sizes in TH listeners.

### Study Limitations

Because our CI population included a relatively small number of participants (n = 18), the current findings may or may not be representative of the CI population at large. Furthermore, any front-end processing algorithms were switched off, and assistive devices other than the CI were removed. These procedures conformed with a controlled laboratory environment and allowed for a reliable assessment of the effect of CIs on the psychometric curve. A risk of these constraints is an underestimated SI, to some extent. Nonetheless, the within-design and age-matched TH group allowed for a robust statistical assessment of the effects of CI use, the differences between speech materials, and the impact of the scoring method on the psychometric curves.

## CONCLUSION

The psychometric curves obtained with the DIN test were the steepest, and those with the Matrix test were the shallowest. The slopes of the psychometric curves obtained from CI users were consistently lower than those of TH listeners. SRTs were lowest for the DIN test and highest for the Matrix test, and CI users had higher SRTs than TH listeners. Despite the shallow slope and high SRT associated with the Matrix test, the effect sizes of the slope and SRT were in between those of the other two tests. For CI users, the DIN test with digit scoring emerged as attractive because of its steep slope and low variance. Scoring method did not substantially affect the slopes and SRTs for the DIN test and LIST sentences, and we can recommend digit and keyword scoring for both tests. For the Matrix test, sentence scoring yielded even shallower slopes and diminished overall scores in the CI group. We therefore recommend word scoring for the Matrix test, especially for CI users.

## ACKNOWLEDGMENTS

The authors are grateful to the study participants for their time and dedication. The authors thank Nicolas Furnon (Advanced Bionics, European Research Center, Hannover, Germany) for technical support.

## Supplementary Material


