# Supplementary material for: Effect of Speech Material and Scoring Method on Psychometric Curves for Cochlear Implant Users and Typical Hearing Listeners
Source: Ear Hear. 2025 Apr 29;46(5):1329–41. doi: 10.1097/AUD.0000000000001672 (PMC12352559; doi:10.1097/AUD.0000000000001672)
Supplement: Supplementary file 1 [file aud-46-1329-s001.pdf]

Supplementary Table 1. Demographics and characteristics of the CI users (N = 18)

ID	Age	Sex	CVC	Etiology	Years CI	CI	Processor
CI01	67	M	90	Unknown, progressive	17	1j	Q90
CI02	61	F	93	Unknown, progressive	23	CII	Q90
CI03	68	M	76	Traumatic? progressive	5	MS	Q90
CI04	62	M	86	Unknown, progressive	4	MS	Q90
CI05	49	F	95	Unknown, progressive	9	MS	Q90
CI06	66	F	93	DFNA9	11	MS	M90
CI07	59	F	96	Sudden deafness	4	MS	Q90
CI08	62	F	96	Familial? Progressive	4	MS	Q90
CI09	65	M	97	Sudden deafness, Meniere’s	4	MS	M90
CI10	59	M	86	Sudden deafness	23	1J	Q90
CI12	62	F	94	Familial? Progressive	5	MS	M90
CI11	75	F	77	Unknown, progressive	6	MS	M90
CI13	71	M	98	Meniere’s, progressive	6	MS	M90
CI14	61	F	79	Sudden deafness	19	1j	Q90
CI15	66	M	95	Familial, Progressive	23	CII	M90
CI16	61	F	76	Sudden deafness	17	1j	M90
CI17	61	F	87	Unknown, progressive	9	MS	Q90
CI18	64	M	91	DFNA9, progressive	4	MS	Q90
Mean<br>SD<br>Median	63<br>6<br>62	10 F<br>8 M	89<br>8<br>92		11<br>7<br>8	12 MS<br>4 1j<br>2 CII	11 Q90<br>7 M90

CVC: consonant-vowel-consonant phoneme score in quiet at 65 dB SPL; DFNA9: Deafness autosomal dominant 9; 1j: HiRes 90K HiFocus 1j; CII: Clarion CII HiFocus II; MS: HiRes 90K HiFocus Mid-Scala
